# Polymethine Dye-Functionalized Nanoparticles for Targeting CML Stem Cells

**DOI:** 10.1016/j.omto.2020.07.007

**Published:** 2020-07-25

**Authors:** Philipp Ernst, Adrian T. Press, Mike Fischer, Vivien Günther, Christine Gräfe, Joachim H. Clement, Thomas Ernst, Ulrich S. Schubert, Jana Wotschadlo, Marc Lehmann, Christoph Enzensperger, Michael Bauer, Andreas Hochhaus

**Affiliations:** 1 Klinik für Innere Medizin II, Abteilung für Hämatologie und Internistische Onkologie, Universitätsklinikum Jena, Am Klinikum 1, 07747 Jena, Germany; 2 Else-Kröner-Forschungskolleg, Jena, Germany; 3 Klinik für Anästhesiologie und Intensivmedizin, Center for Sepsis Control and Care (CSCC), Universitätsklinikum Jena, Am Klinikum 1, 07747 Jena, Germany; 4 Jena Center for Soft Matter (JCSM), Friedrich Schiller Universität Jena, Philosophenweg 7, 07743 Jena, Germany; 5 Laboratory of Organic and Macromolecular Chemistry (IOMC), Friedrich Schiller Universität Jena, Humboldtstrasse 10, 07743 Jena, Germany; 6 SmartDyeLivery GmbH, Botzstrasse 5, 07743 Jena, Germany

**Keywords:** polymethine dyes, nanoparticles, CML, OATP1B3, OCT1

## Abstract

In chronic myelogenous leukemia (CML), treatment with tyrosine kinase inhibitors (TKI) is unable to eradicate leukemic stem cells (LSC). Polymethine dye-functionalized nanoparticles can be internalized by specific cell types using transmembrane carrier proteins. In this study we investigated the uptake behavior of various polymethine dyes on leukemia cell lines and searched for carrier proteins that guide dye transport using RNA interference. The results show that the uptake of DY-635 is dependent on organic anion transport protein 1B3 (OATP1B3) in CML cells and immature myeloid precursor cells of CML patients. In contrast to nonspecific poly(lactide-*co*-glycolic acid) (PLGA) nanoparticle constructs, DY-635-functionalization of nanoparticles led to an uptake in CML cells. Investigation of these nanoparticles on bone marrow of CML patients showed a preferred uptake in LSC. The transcription of OATP1B3 is known to be induced under hypoxic conditions via the hypoxia-inducing factor 1 alpha (HIF1α), thus also in the stem cells niche. Since these cells have the potential to repopulate the bone marrow after CML treatment discontinuation, eliminating them by means of drug-loaded DY-635-functionalized PLGA nanoparticles deployed as a selective delivery system to LSC is highly relevant to the ongoing search for curative treatment options for CML patients.

## Graphical Abstract

**Figure:**
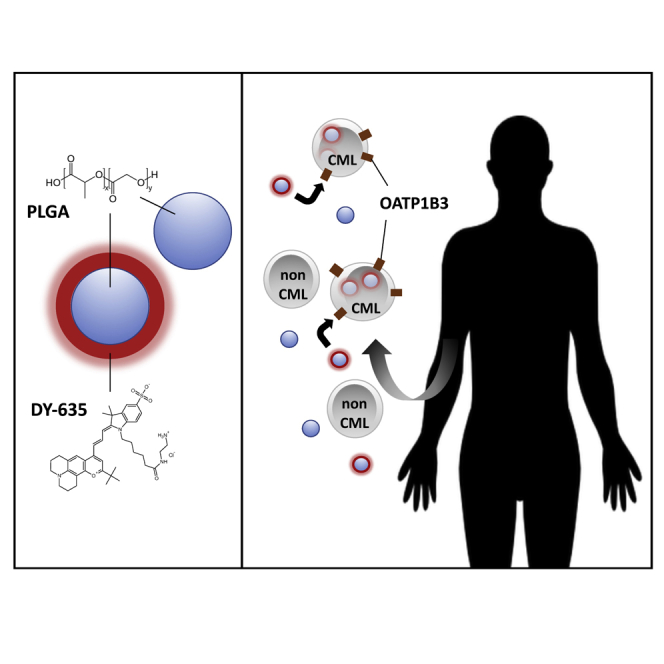

## Teaser

With the finding that DY-635-functionalized nanoparticles are taken up *in vitro* in an OATP1B3-dependent manner, Ernst et al. identified a possible drug delivery system for CML treatment. This would be an important advance, since TKIs are ineffective in CML stem cells, but their eradication is necessary for curative treatment.

## Introduction

Chronic myelogenous leukemia (CML) is a myeloproliferative neoplasia caused by the translocation t(9;22)(q34;q11) in the pluripotent hematopoietic stem cell with formation of a constitutively active *BCR-ABL1* tyrosine kinase.^1^ Due to its monogenetic origin with the option of single drug tyrosine kinase inhibitor (TKI) therapy, CML is considered to be the model of targeted therapy of malignant disease. However, treatment with TKIs is unable to eradicate leukemic stem cells (LSCs) in CML patients. These cells have the potential to repopulate the bone marrow, leading to relapse.^2^ Similar to normal hematopoietic stem cells (HSCs), LSCs have the ability to self-renew and establish a state of quiescence.^2^^,^^3^ Since CML stem cells suppress *BCR-ABL1* expression under treatment with TKIs, tyrosine kinase-independent mechanisms such as changes in mitochondrial metabolism, epigenetic modifications, and alterations of the transcriptional regulatory networks maintained by the stem cell niche are responsible for LSC persistence.^4^^,^^5^ Imatinib and other TKIs targeting *BCR-ABL1*, as well as established chemotherapeutic drugs, inhibit cell proliferation, thereby inducing apoptosis. Therefore, they are not effective against non-proliferating progenitor cells and stem cells.^6^ New, innovative and gentler treatment strategies to eliminate the remaining CML stem cells must be developed to completely cure CML patients who achieve a good molecular response after initial TKI therapy.

Poly(lactide-*co*-glycolic acid) (PLGA) is a US Food and Drug Administration (FDA)-approved biodegradable copolymer that degrades *in vivo* through lysosomal hydrolysis of its ester bonds to lactate and glycolate, which are finally metabolized to CO_2_ and H_2_O.^7^ The degradation to non-toxic products qualifies PLGA nanoparticles for clinical applications.^8^ Recently it was shown that encapsulation of TLR 7/8 bi-specific agonists in PLGA nanoparticles lead to an anticancer immunostimulation when applied in melanoma, bladder, and renal cell carcinoma tumor models.^9^ In the form of a delivery system for WDVAX, an injectable cancer vaccine, PLGA is currently being tested in a phase 1 trial in metastatic melanoma patients for the first time (NCT01753089).^10^ Modifications on the surface of nanoparticles possess the property of being more strongly and to a certain degree more selectively internalized by different tissues. For example, the delivery of paclitaxel by anti-HER2/neu peptide-conjugated iron oxide nanoparticles to HER2/neu-overexpressing breast cancer cells has been demonstrated in a mouse model.^11^ Furthermore it was shown that PLGA nanoparticles functionalized with a polymethine dye shell can be selectively internalized by specific tissues due to their affinity for transmembrane carrier proteins.^12^ The cationic nanoparticles thus functionalized can transport active ingredients and are internalized by the target cell via clathrin-mediated endocytosis.^13^ It has been established that hydrophobic polymethine dyes are taken up by hepatocytes via a pattern of carrier proteins, especially organic anion transport proteins (OATP1B1, OATP1B3) and organic cation transporters (OCT1).^12^ Since CML cells mainly use OCT1 and OATP1B3 for the uptake of imatinib,^14^ it is important to determine whether a dye uptake behavior similar to that of hepatocytes can be observed in CML cells. If indeed a similarity can be established, it is conceivable that dye-functionalized nanoparticles could be used as a selective drug delivery system for CML cells, in particular for CML stem cells.

In this study, we investigated four chemically related polymethine dyes and their uptake behavior in CML and AML cell lines, as well as in MNCs from 30 patients with newly diagnosed and untreated CML. After incubating the cells, flow cytometry and confocal laser scanning microscopy were performed to analyze the quantitative uptake and the dye localization in the cells. In addition, quantitative real-time PCR was performed to determine expression levels of mRNA coding for various carrier proteins that are known to be important for the clathrine-mediated uptake of polymethine dyes. Subsequently, knockdown experiments were done to investigate whether the dye uptake is mediated by a particular carrier protein. PLGA nanoparticles with a Nile Red core were then synthesized and covalently conjugated with a specific polymethine dye shell in order to determine whether the functionalization of the nanoparticles improves their uptake in comparison to non-functionalized nanoparticles.

## Results

### Uptake Behavior of Related Polymethine Dyes Differ from Each Other

**Figure 1:**
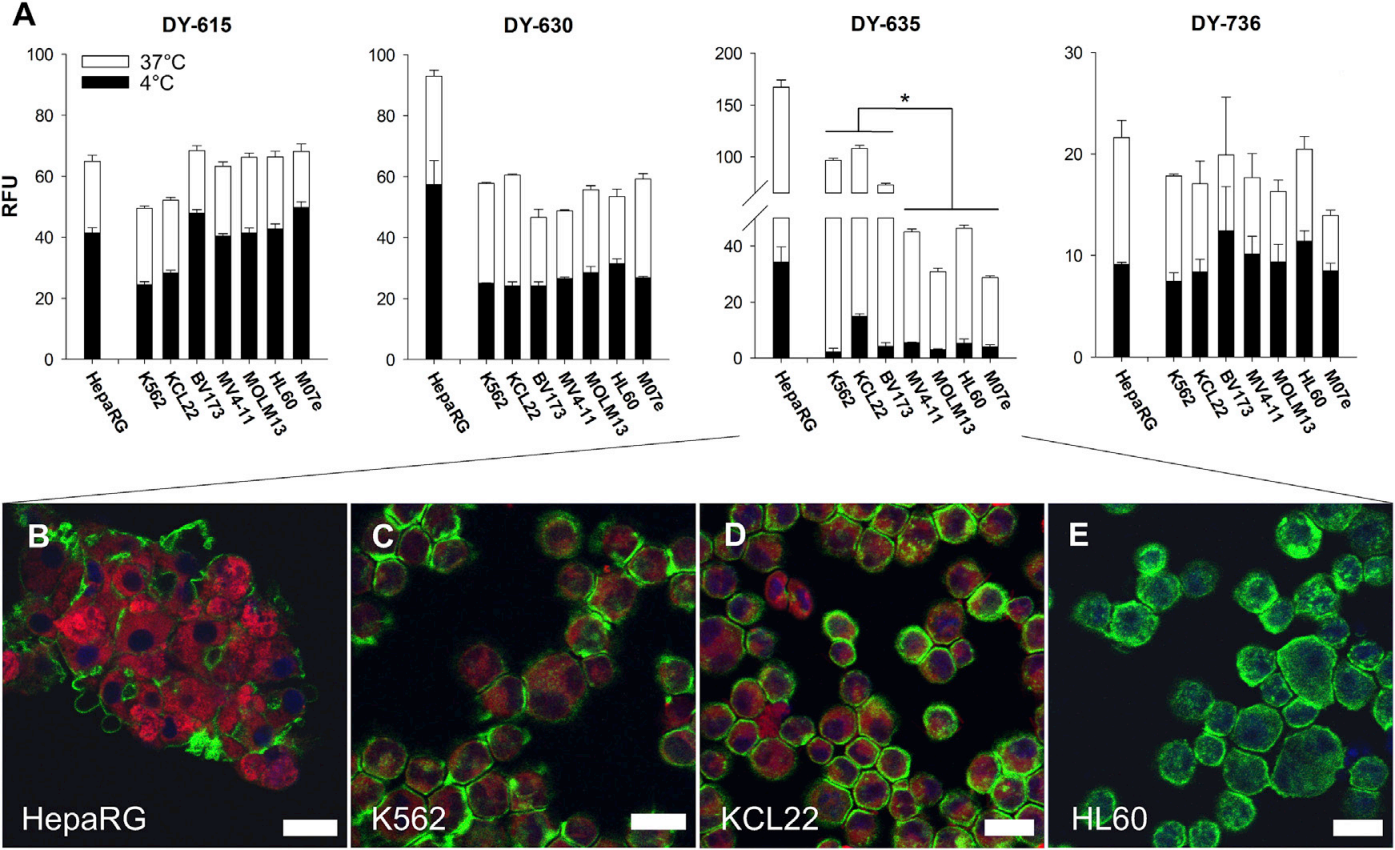
DY-635 Shows Different Uptake Behavior Dependent on the Cell Line (A) Flow cytometric determined relative fluorescence units (RFUs) after incubation of 100 nM DY-615, DY-630, DY-635, and Dy-736 with HepaRG cells, CML cell lines (K562, KCL22, BV173) and AML cell lines (MV4-11, MOLM13, M07e). Shown are mean values with their standard errors. ∗p < 0.05. (B–E) Laser scanning micrographs of HepaRG cells (B), K562 cells (C), KCL22 cells (D), and HL60 cells (E) after incubation with 100 nM DY-635 (red). Cytoskeleton staining with phalloidin Alexa 488 (green). Cell nucleus staining with DAPI-II (blue). Scale bars, 20 μm.

The cellular dye uptake of four polymethine dyes was studied. DY-615, DY-630, DY-635, and DY-736 were selected on the basis of their physicochemical properties. Dye incubation was carried out on HepaRG cells, on CML cell lines K562, BV173, and KCL22, as well as on the AML cell lines MV4-11, MOLM13, HL60, and M07e. DY-736, DY-615, and DY-630 showed no significant difference between CML and AML cell lines (RFU DY-736 18.3 ± 1.5 versus 17.1 ± 2.7, p = 0.95; RFU DY-615 56.7 ± 10.2 versus 66.0 ± 2.0, p = 0.33; RFU DY-630 55.0 ± 7.4 versus 54.3 ± 4.4; p = 0.47). Furthermore, the passive uptake of these dyes was relatively higher when incubated at 4°C compared to 37°C (DY-736 53.4% ± 8.8%, DY-615 67.0% ± 12.0% and DY-630 50.9% ± 9.2%). Of all dyes studied, DY-635 revealed the strongest fluorescence signal after incubation with HepaRG cells, as well as a significantly higher uptake by CML cells than by AML cells (RFU 92.5 ± 18.1 versus 37.7 ± 9.2; p = 0.023; Figure 1A). Also, passive uptake of DY-635 was lowest at 4°C compared to 37°C (11.8% ± 6.3%). Confocal laser scanning microscopic analysis of dye uptake by both the HepaRG cells and the suspension cell lines showed that the polymethine dyes were detectable only in the cytoplasm without staining of the nucleus (Figures 1B–1D). DY-635 had a homogeneous distribution over the entire cytoplasm in the cell cluster. The analysis of the dye uptake of CML cell lines showed an increased enrichment of DY-635 in KCL22 cells compared to K562 cells, analogous to the flow cytometry results (Figures 1C and 1D).

### DY-635 Uptake Depends on the Presence of OATP1B3

**Figure 2:**
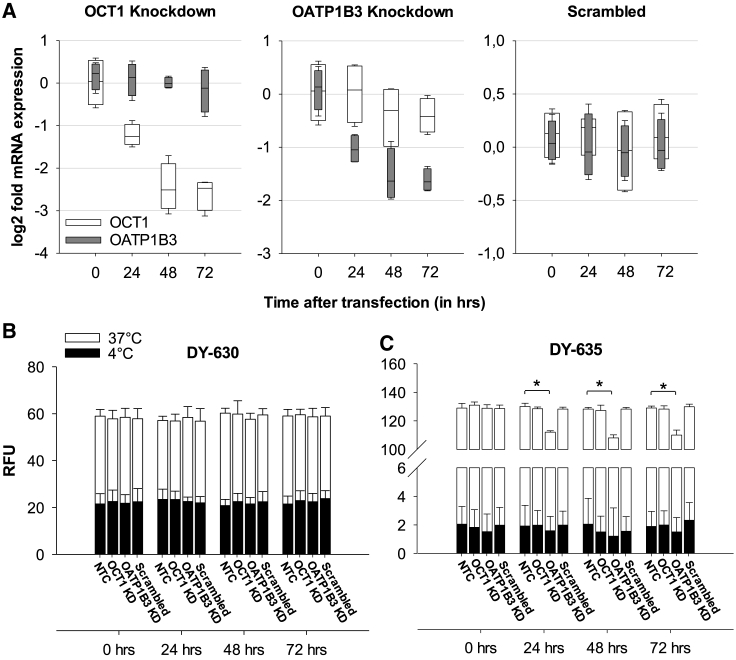
DY-635 Uptake Depends on the Presence of OATP1B3 (A) Quantitative real-time PCR of mRNA expression of OCT1 and OATP1B3 genes after siRNA knockdown in K562 cells. (B and C) Uptake of 100 nM DY-630 (B) and DY-635 (C) from both non-transfected K562 cells (NTCs) and K562 cells immediately before and 24, 48, and 72 h after transfection with OCT1, OATP1B3, or scrambled siRNA. KD, knockdown. RFU, relative fluorescence units. ∗p < 0.05.

Since the uptake of polymethine dyes appears to depend on the presence of certain carrier proteins, mRNA expression of the genes coding for the influx-proteins OATP1B1, OATP1B3, OCT1, and NTCP, as well as for the efflux-protein ABCB1 was determined by quantitative real-time PCR in all cell lines and correlated with the quantitative dye uptake of each cell line. An extremely low to undetectable mRNA expression of OATP1B1 and NTCP was found in all cell lines compared to the expression level in HepaRG cells. The mRNA expression of OATP1B3 was variable in the suspension cell lines, but on average 27.8-fold lower than in the HepaRG cells. The mRNA expression of OCT1 in the CML and AML cell lines was on average 45.2-fold lower than the HepaRG cells and showed a smaller range of variation among the suspension cells than OATP1B3 (Figure S2). Using a Pearson correlation, a significant relationship was observed between the DY-635 uptake and the corresponding OATP1B3 expression level (R = 0.71, p = 0.02) and between the DY-630 uptake and the corresponding ABCB1 expression level (R = 0.88, p = 0.004; Figure S3). To detect a causal relationship between the presence of influx proteins and dye uptake, we transiently downregulated OATP1B3 and OCT1 by small interfering RNA (siRNA) transfection in the CML cell line K562 (Figure 2A). 72 h after transfection, K562 cells with an OATP1B3 knockdown showed a significantly lower uptake of DY-635 compared to the wild-type (RFU 110.0 ± 3.6 versus 129.0 ± 1.3, p = 0.001). OCT1 knockdown showed no change in fluorescence behavior (Figure 2C). After incubation with DY-630, no difference in fluorescence was observed between K562 cells with or without knockdown of OCT1 and OATP1B3, respectively (Figure 2B).

### Immature CML Cells Take Up DY-635 in a Dominant Manner

**Figure 3:**
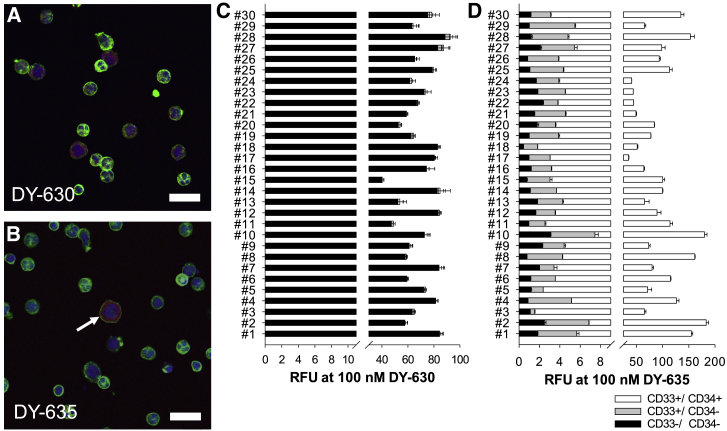
Immature Precursor Cells of CML Patients Prefer DY-635 Uptake (A and B) Laser scanning microscopic analysis of mononuclear cells of a patient with newly diagnosed and untreated CML after incubation with 100 nM DY-630 (red in A) and 100 nM DY-635 (red in B). Immature precursor cell (arrow in B). Cytoskeleton (green, phalloidin Alexa 488). Nucleus (blue, DAPI-II). Scale bars, 20 μm. (C and D) Flow cytometric results from CD33^−^/CD34^−^ (black) and CD33^+^/CD34^−^ (dark gray) and CD33^+^/CD34^+^ (white) cells from 30 patients with newly diagnosed and untreated CML after incubation with 100 nM DY-630 (C) and DY-635 (D). Shown are mean values with their standard deviation. RFU, relative fluorescence units.

**Figure 4:**
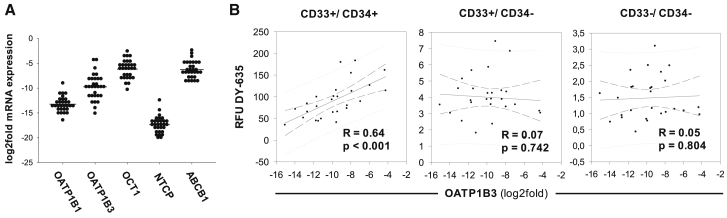
OATP1B3 Expression of Immature Precursor Cells Is Associated with DY-635 Uptake in CML Patients (A) Density plots of the carrier protein expression in leukocytes from 30 CML patients. The mRNA expression of carrier proteins is shown as log2fold mRNA expression relative to the expression level of HepaRG cells. The horizontal line marks the mean value. (B) Pearson correlation of the RFUs of DY-635 of the cell fractions CD33^+^/CD34^+^, CD33^+^/CD34^−^, and CD33^−^/CD34^−^ and the log2fold mRNA expression of OATP1B3. N = 30.

In addition to the *in vitro* cell lines, dye uptake behavior was performed *ex vivo* on the cells of 30 newly diagnosed CML patients. Extracted MNCs were incubated with DY-630 and DY-635 and labeled with fluorophore-conjugated CD33 and CD34 antibodies. In all patients, the mean distribution of CD33^−^/ CD34^−^, CD33^+^/CD34^−^, and CD33^+^/CD34^+^ cell populations were 52.2% ± 11.0%, 41.7% ± 8.5%, and 5.5% ± 3.0%, respectively. After incubation with 100 nM DY-630, there was no detectable difference in the uptake behavior of the three cell populations (CD33^−^/CD34^−^: RFU 68.7 ± 12.6; CD33^+^/CD34^−^: RFU 68.7 ± 13.1; CD33^+^/CD34^+^: RFU 69.6 ± 13.0, one-way ANOVA, p = 0.93; Figure 3C). Laser scanning microscopy confirmed the uptake of DY-630 in both myeloid and lymphatic cells (Figure 3A). In contrast, after incubation with 100 nM DY-635, the CD33^+^/CD34^+^ cells showed a dominant dye uptake and the CD33^+^/CD34^−^ and CD33^−^/CD34^−^ cells a low uptake (CD33^−^/CD34^−^: RFU 1.43 ± 0.6; CD33^+^/CD34^−^: RFU 4.0 ± 1.3; CD33^+^/CD34^+^: RFU 94.3 ± 41.9, one-way ANOVA, p < 0.001; Figure 3D). Analogous to the flow cytometric data, laser-scanning microscopy found an isolated accumulation of DY-635 in cells that appeared morphologically immature due to their large cell size, their roundish nucleus, and the high nuclear-plasma ratio (Figure 3B). The leukocyte mRNA expression of OATP1B1, OATP1B3, OCT1, NTCP, and ABCB1 of these CML patients was also measured by quantitative real-time PCR (Figure 4A) and correlated with the dye uptake behavior of the three cell fractions CD33^−^/CD34^−^, CD33^+^/CD34^−^, and CD33^+^/CD34^+^ after incubation with DY-630 and DY-635. This method revealed a significant correlation between the DY-635 uptake in CD33^+^/CD34^+^ myeloid progenitor cells and the corresponding OATP1B3 expression level (Figure 4B).

### Functionalization of PLGA Nanoparticles with DY-635 Enables Entry into CML Cells

**Figure 5:**
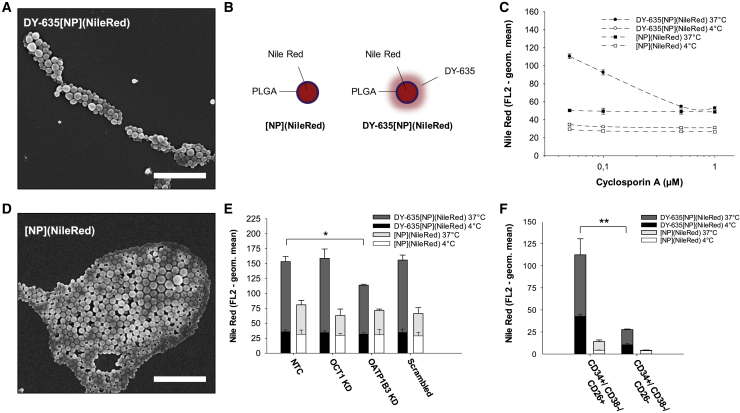
Functionalization of PLGA Nanoparticles with DY-635 Increases Nanoparticle Uptake in CML Cells with Stem Cell Signature (A) SEM of DY-635 functionalized PLGA nanoparticles DY-635[NP](NileRed). Scale bar, 2 μm. (B) Schematic illustration of non-functionalized nanoparticles [NP](NileRed) and DY-635[NP](NileRed) both labled with a Nile Red core. (C) Flow cytometric results of nanoparticle incubation of K562 cells with competitive antagonism of cyclosporin A. (D) SEM of [NP](NileRed). Scale bar, 2 μm. (E) Nanoparticle uptake of NTCs and 72 h after OCT1, OATP1B3, and scrambled siRNA transfection, respectively. Shown are mean values with their standard errors. KD, knockdown. (F) Flow cytometric results of nanoparticle incubation of CML cells with stem cell signature (CD34^+^/CD38^−^/CD26^+^) and hematopoietic stem cells (CD34^+^/CD38^−^/CD26^−^). N = 5. ∗p < 0.05. ∗∗p < 0.01.

Based on the observation that DY-635 uptake is OATP1B3-dependent, it was further investigated whether the covalent linkage of DY-635 to PLGA nanoparticles leads to improved nanoparticle uptake in CML cells. Therefore, DY-635-conjugated PLGA nanoparticles *DY-635[NP](NileRed)* were synthesized and marked with Nile red (Figures 5A and 5B). Non-functionalized nanoparticles without a polymethine dye shell *[NP](NileRed)* were used as negative control (Figures 5B and 5D). After 30 min incubation of 5 × 10^5^ K562 cells at 37°C with both 50 μg mL^-1^ *[NP](NileRed)* and *DY-635[NP](NileRed)*, a 2.7-fold increase in fluorescence intensity was detected after incubation with *DY-635[NP](NileRed)* compared to *[NP](NileRed)* (Geometric Mean FL2 138.4 ± 2.9 versus 50.8 ± 3.7). Also, a 3.9-fold increase in the cellular *DY-635[NP](NileRed)* uptake at 37°C was observed compared to the passive diffusion of *DY-635[NP](NileRed)* at 4°C (Geometric Mean FL2 138.4 ± 2.9 versus 35.5 ± 1.5). Incubating of the nanoparticles with increasing concentrations of cyclosporin A, an FDA-approved OATP inhibitor, resulted in a gradual reduction of the cellular *DY-635[NP](NileRed)* uptake of 94.9% ± 3.2% at 0.1 μM cyclosporin A up to 50.5% ± 6.0% at 1.0 μM cyclosporin A in the sense of a competitive antagonism (Figure 5C). After incubation of equimolar concentrations of the two nanoparticle constructs with K562 cells 72 h after knockdown of OCT1 and OATP1B3, the quantitative uptake of the nanoparticles was determined by flow cytometry using the fluorescence intensity of Nile Red (FL2 channel). Similar to the dye incubation experiments, OATP1B3 knockdown significantly reduced the uptake of *DY-635[NP](Nile Red)* in K562 cells compared to wild-type cells (geometric mean 114.0 ± 1.7 versus 153.5 ± 8.7, p = 0.001 and 114.0 ± 1.7 versus 158.9 ± 15.7, p = 0.008; Figure 5E). No significant difference in the quantitative uptake of *[NP](NileRed)* was detected between wild-type K562 cells and cells with OCT1 or OATP1B3 knockdown.

### DY-635 Functionalized PLGA Nanoparticles Prefer Leukemic Stem Cells in CML

Having demonstrated that functionalization of PLGA nanoparticles with DY-635 sensitizes nanoparticles for CML cells and that DY-635 seems to be taken up predominantly by myeloid precursor cells in CML patients, we investigated further to determine whether DY-635-functionalized PLGA nanoparticles could distinguish between HSCs (CD34^+^/CD38^−^/CD26^−^) and LSCs (CD34^+^/CD38^−^/CD26^+^) in CML patients. Therefore, both nanoparticle constructs *DY-635[NP](NileRed)* and *[NP](NileRed)* were incubated with selected CD34-positive bone marrow cells from five patients with newly diagnosed CML in chronic phase that were additionally labeled with fluorophore conjugated CD38 and CD26 antibodies. We successfully demonstrated a significantly higher uptake of *DY-635[NP](NileRed)* by LSC compared to *[NP](NileRed)* (RFU 112.3 ± 18.1 versus 14.2 ± 1.8, p = 0.009; Figure 5F).

## Discussion

The primary objective of this study was to study polymethine dye-functionalized nanocarrier systems as a novel entry into leukemia cells. To do so, we investigated whether the internalization of the dyes and the dye-functionalized nanoparticles is determined by certain carrier proteins. The data show that DY-635-uptake is significantly higher in CML cells than in AML cells and that the uptake correlates with the expression level of OATP1B3 in CML cells. A heterologous expression system using HEK293 cells revealed a high affinity of DY-635 to OATP1B1 and OATP1B3.^12^ This study confirmed this relationship by a knockdown of OATP1B3 in K562 cells and, consequently, a significantly lower uptake of DY-635 compared to wild-type.

With the exception of the basolateral hepatocyte membrane where OATPs are typically localized, a higher expression of OATP1B3 has been detected in various tumor entities, e.g., colorectal, prostate, gastric, urinary, pancreatic, ovarian, and thyroid carcinomas, than in the corresponding healthy organ tissue.^15^^,^^16^ It is known that tumor cells operate only to a small extent with mitochondrial respiration and that they increase anaerobic glycolysis in order to adapt their energy production to the oxygen-poor environment.^17^ An important factor for maintaining this metabolic pathway is the hypoxia-inducible factor 1 (HIF1). Under hypoxic conditions, the HIF1α subunit remains stable, leading to the upregulation of genes that have hypoxia response element (HRE) binding sites for HIF1α in their promoters.^18^ The glycolysis and neoangiogenesis are typically regulated in this way.^18^^,^^19^ Wu et al.^20^ demonstrated that the interaction of HIF1α with the HRE of the promoter of the *OATP1B3* gene under hypoxic conditions leads to an increased transcription of OATP1B3. In addition to the transport of imatinib, OATP1B3 is a carrier protein for various chemotherapeutic agents such as paclitaxel, docetaxel, irinotecan, and methotrexate.^21^ The OATP1B3-mediated uptake of polymethine dyes in tumor cells has already been established for near-infrared heptamethine carbocyanines such as MHI-148, IR-780, IR-783, and IR-808.^20^^,^^22^^,^^23^ These heterocyclic polymethine cyanins are predominantly incorporated into lysosomes and mitochondria of various types of tumor cells, including cultured, circulating, and disseminated tumor cells, but not in healthy tissue.^24^, ^25^, ^26^ In mouse models, heptamethine dyes have already been used as delivery systems in tumor therapy.^23^ Wu et al.,^27^ for example, were able to achieve a significant reduction of osseous metastases of a prostate carcinoma by applying an IR-783-docetaxel conjugate in the mouse model. Similar to the heptamethine dyes shown here, the affinity of DY-635 to OATP1B3 on CML cells could be used as a delivery system for therapeutic applications.

There is increasing evidence that TKI therapy achieves a time-dependent deep molecular response in nearly 40% of patients, which suggests that at least a temporary discontinuation of TKIs may be feasible.^28^ So far, only a small subgroup of patients has achieved a successful treatment-free remission of more than 5 years.^29^ The biological cause for a relapse is located in quiescent LSC, which cannot be eliminated by TKIs or conventional chemotherapeutic drugs.^2^^,^^3^ In this study, the incubation of DY-635 with mononuclear cells from patients with newly diagnosed CML showed a nearly selective uptake of the polymethine dye by a CD33^+^/CD34^+^ cell fraction. These cells are a mixed population of multipotent progenitor cells (Lin^−^/CD34^+^/CD38^+^/CD45RA^−^/CD90^+^), which already have a myeloid differentiation by coexpression of CD33.^30^ Earlier hematopoietic stem cells (Lin^−^/CD34^+^/CD38^−^/CD45RA^−^/CD90^+^) exhibit long-term, multilineage repopulating ability and are found almost exclusively in the bone marrow.^30^ A significant proportion of the early progenitor cells in the peripheral blood of CML patients is determined not by normal but by leukemic progenitors, since these have a higher proliferative capacity than normal progenitors.^31^ Immunophenotypically, the CML blasts can be distinguished from normal hematopoietic stem cells by the co-expression of dipeptidylpeptidase IV (DPPIV, CD26).^32^ The hypoxic niche of the bone marrow has an oxygen content of <1%, which appears to regulate hematopoiesis by maintaining important hematopoetic stem cell functions.^33^^,^^34^ Zang et al.^35^ demonstrated that HIF1α is highly regulated in the *BCR-ABL1*-expressing LSCs and is necessary for the survival of the LSCs in CML. As previously mentioned, solute carrier organic anion transporter family member 1B3 (*SLCO1B3*) is a target gene of HIF1α. It can therefore be assumed that OATP1B3 is expressed more frequently in the CML LSCs than in the normal HSCs. Nies et al.^36^ compared the mRNA expression of different SLC drug transporters in primary CD34^+^ cells within a small cohort of Ph^+^ CML patients with Ph^−^ non-CML donors. Similar to our study, a low level of *SLCO1B3* expression was detected in both groups. A statistically significant difference in the expression of *SLCO1B3* in CD34^+^ cells from CML patients and in Ph^−^ non-CML donors was not detectable.^36^ In this work, a positive correlation was found between the expression of OATP1B3 in CD33^+^/CD34^+^ cells and the intensity of the DY-635 uptake.

We were able to show that the functionalization of PLGA nanoparticles with DY-635, *DY635[NP](NileRed)*, leads to an increased internalization in CML cells compared to the native PLGA nanoparticles, *[NP](NileRed)*. As in the incubation with the dye, a knockdown of OATP1B3 in CML cells resulted in a significantly lower uptake of *DY635[NP](NileRed)*, suggesting a dependence on this carrier protein. As mentioned above, the dye incubation trial with MNCs from CML patients revealed that DY-635 appears to be taken up predominantly by early progenitor cells. To determine whether this uptake is characteristic of LSCs or both LSCs and HSCs, we isolated CD34-positive cells from bone marrow of five CML patients and categorized them immunophenotypically into CD38^−^/CD26^+^ LSCs and CD38^−^/CD26^−^ HSCs. We were thus able to demonstrate that nanoparticle functionalization with DY-635 leads to a significantly higher and apparently selective internalization in LSCs of CML patients. Given the fact that PLGA nanoparticles can be used as a delivery system for drugs, such functionalization would allow to specifically target and eliminate persistent LSCs from CML patients in molecular remission. It is well-known that quiescent LSC in CML cannot be eradicated by TKI treatment. In contrast to established cytostatic drugs, more selective compounds should be considered for encapsulation in DY635-functionalized PLGA nanoparticles in order to avoid side effects. Recently it has been shown that *in vivo* CRISPR-Cas9 genome editing can be achieved using simultaneous delivery of Cas9 mRNA and sgRNA via bioreducible lipid nanoparticles.^37^ In the case of DY635-functionalized PLGA nanoparticles, it would be beneficial to package Cas9 mRNA with sgRNAs, that complementary targets the individual *BCR-ABL1* breakpoint of CML patients. This would make the development of an even more precisely targeted therapy for the eradication of remaining CML stem cells possible.

## Materials and Methods

### Cell Lines and Patients

For cell culture examinations, CML cell lines K562, BV173, KCL22, AML cell lines HL60, MV4-11, MOLM13, M07e (German Collection of Cell Cultures (DSZM), Braunschweig, Germany) and the hepatic stem cell line HepaRG (Thermo Fisher Scientific, Waltham, MA, USA) were used. The suspension cell lines were cultured in 90% RPMI medium (Invitrogen GmbH, Karlsruhe, Germany) with 10% fetal calf serum (FCS) (Thermo Fisher Scientific, Darmstadt, Germany). For M07e cells, 10 ng mL^-1^ GM-CSF was added to the culture medium. The complete culture medium was renewed every 2 to 3 days. All cell lines were regularly tested for *mycoplasma spp* with PCR-based assay. The adherent HepaRG cells were grown on cover slides in 24-well plates in William’s Medium E (Biochrom, Berlin, Germany), 100 IU mL^-1^ penicillin, 100 μg mL^-1^ streptomycin, 2 mmol L^-1^ L-glutamine (Thermo Fisher Scientific, Paisley, UK), and 10 μg mL^-1^ human insulin (Sigma-Aldrich, Darmstadt, Germany). The complete culture medium was renewed every 3 to 4 days and, each time, 5 μmol L^-1^ hydrocortisone hemisuccinate (Sigma-Aldrich) was added. After reaching 90% confluency, cell growth was stopped by the addition of 2% (v/v) dimethyl sulfoxide (DMSO; Carl Roth, Karlsruhe, Germany) and cell differentiation was induced. Over a period of 3 to 4 weeks HepaRG cells matured into a mixed population of hepatocyte-like colonies surrounded by biliary-like epithelial cells and were then used for experiments.

30 patients with newly diagnosed and untreated *BCR-ABL1*-positive CML were investigated. The study was approved by the local ethics committee and all patients provided their written informed consent. Median age of patients was 53 years (range, 19 to 74 years), 16 harbored the *BCR-ABL1* transcript b3a2, 11 patients b2a2, and three patients both b3a2 and b2a2. Peripheral blood was taken from 25 patients and both peripheral blood and bone marrow were taken from 5 patients at diagnosis (Table S1).

### MNC Extraction and CD34 Separation

For the extraction of mononuclear cells (MNCs), peripheral blood or bone marrow was incubated with RPMI medium 1:2 to max. 25 mL and carefully added to 15 mL Biocoll. This was then centrifuged for 40 min at 400 × *g* (Eppendorf 5810R). After transfer of the MNCs into a 50 mL Falcon tube, it was filled with RPMI medium and centrifuged at 400 × *g* for 10 min at 4°C. The supernatant was discarded and the tube refilled with RPMI medium and again centrifuged for 10 min at 400 × *g* at 4°C. The pellet was subsequently resuspended in 5 mL RPMI medium. The CD34^+^ separation was carried out with the MACS® Magnetic MicroBead Technology (CD34 MicroBead Kit Ultra Pure, human, Miltenyi Biotec). The MNC pellet was resuspended with 300 μL of PE buffer (phosphate buffered saline [PBS] with ETDA) and incubated with 100 μL of blocking reagent and CD34 beads in equal parts for 90 min at 4°C on a shaker. The tube was then filled with PE buffer and centrifuged for 5 min at 200 × *g* at 4°C. After equilibration of the magnetic column with 1 mL of PE buffer, 5 mL of cell suspension were passed through the magnetic column and the flow was discarded. The magnetic column was washed twice with PE buffer. CD34-positive cells were eluted with 1 mL of PE buffer. After the cell count was determined, cells were centrifugated for 5 min at 200 × *g* at 4°C and the supernatant was discarded.

### Polymethine Dyes and Nanoparticle Synthesis

DY-615, DY-630, DY-635, and DY-736 synthesized by Dyomics (Dyomics GmbH, Jena, Germany) were used in our experiments (Table S2). The nanoparticles used were based on a polymer matrix of PLGA (SmartDyeLivery GmbH, Jena, Germany). In order to enable the detection of the nanoparticles by flow cytometry and fluorescence microscopy, the particles were given a core of a fluorescent Nile Red dye (Sigma-Aldrich). Nanoparticle formulations were prepared by nanoprecipitation from PLGA with covalently bound DY-635 and Nile Red as payload (*DY-635[NP](NileRed)*) and from pure PLGA loaded with Nile Red (*[NP](NileRed)*; Figures 5A, 5B, and 5D). After preparation, nanoparticles were purified via centrifugation for 45 min at 16,000 × *g* at 4°C. The supernatant was discarded completely and the pellet was resolved in 0.5% sucrose solution. The working solution of mentioned nanoparticle formulations was diluted to a concentration of 100 nM with double distilled water. After synthesis of *DY-635[NP](NileRed)* and purification processes, no free DY-635 molecules were found via size exclusion chromatography (SEC). The hydrodynamic diameters were determined as size values by dynamic light scattering (DLS) and the surface charge of the nanoparticles by zeta potential measurements with a Zetasizer Nano ZS (Malvern Panalytical) with 173° back scattering angle (Figure S1). The particle size values were determined by intensity and the standard setting for polymer nanoparticles were used for analysis (dispersant: water; refractive index: 1.33; dynamic viscosity 0.8872 mPa × s; dielectric constant 78.5). Five measurements were performed after an equilibration time of 30 s at 25°C. Three zeta potential measurements were performed with a zeta cell DTS1070, each with 20 subruns. Before measuring hydrodynamic diameter and zeta potential, nanoparticle suspensions were diluted with double distilled water 1:10. After preparation, nanoparticles were resolved in double distilled water with 0.5% sucrose. The mean hydrodynamic diameters, calculated based on intensities from DLS measurements, were 207 nm (*DY-635[NP](NileRed)*; Sample SDL-004-050-01) and 241 nm (*[NP](NileRed)*; Sample SDL-004-048-01; Table S3). A polydispersity index (PDI) of 0.077 and 0.119 indicates a narrow distribution of the particle size. Nile Red and DY-635 concentration in the nanoparticle suspensions was determined by absorption and fluorescence measurements with a multiplate reader at 552 and 658 nm, respectively (Table S4).

### Dye and Nanoparticle Application Experiments

From each cell line and from the freshly isolated MNCs of the CML patients, 100 μL containing 5 × 10^5^ cells were pipetted into 1.5 mL tubes in duplicate and stored at 37°C in a thermocycler (Thermomixer comfort, Eppendorf) or at 4°C on ice for at least 10 min. The cells were incubated in parallel at 37°C and 4°C with 100 μL of the different dye concentrations (1 nM, 3 nM, 10 nM, 30 nM, and 100 nM) for 5 min in the dark and then fixed for 15 min with 10% formalin solution. In the case of MNCs, a further washing step with D-PBS + 1% BSA was followed by incubation of 50 μL each with phycoerythrin (PE)-conjugated anti-CD33 antibodies (BD Biosciences) and fluorescein isothiocyanate (FITC)-conjugated anti-CD34 antibodies (BD Biosciences) in a 1:5 dilution over 15 min in the dark. The samples were centrifuged at 100 × *g* for 5 min (Eppendorf Centrifuge 5415 C) and washed twice with 1 mL D-PBS + 1% BSA after discarding the supernatant. The pellet was resuspended in 500 μL D-PBS + 1% BSA, transferred to fluorescence-activated cell sorting (FACS) tubes and measured after short vortexing using a BD FACSCalibur system.

For nanoparticle uptake experiments, 100 μL with 5 × 10^5^ K562 cells per well were seeded in 24-well plates in duplicate for incubation experiments at 4°C and 37°C, respectively. First increasing concentrations of cyclosporin A (0.05 μM, 0.1 μM, 0.5 μM, 1.0 μM) were incubated. 5 min later, 100 μL of prediluted solutions from *[NP](NileRed)* and *DY-635[NP](NileRed)* were added in the dark each to obtain incubation concentrations of 50 μg mL^-1^ per well. 30 min later, the cells were fixed with 10% formalin solution for 20 min at room temperature (RT). Subsequently, the cell suspensions were transferred into 1.5 mL tubes and washed three times with D-PBS. Finally, the cells were transferred into FACS tubes with 500 μL of D-PBS and immediately measured with the flow cytometer. For the investigation of the nanoparticle uptake of K562 cells with knockdown of the carrier proteins OCT1 and OATP1B3 72 h after siRNA application (see RNA Interference), an aliquot of 1 × 10^5^ cells was removed from the corresponding wells in a volume of 100 μL RPMI + 10% FCS in 1.5 mL tubes. The cells were then incubated for 30 min in the dark at 4°C and 37°C in the presence of 10 μg mL^-1^ *[NP](NileRed)* or *DY-635[NP](NileRed)*. The cells were washed three times with D-PBS and then transferred to FACS tubes for measurement with the flow cytometer.

### Flow Cytometry

Flow cytometric data were evaluated using FlowJo vX.0.7. Annexin V-antigen-presenting cell (APC)/propidium iodide assay was used as vitality verification. For every measurement, 2 × 10^4^ events were analyzed in duplicate. To analyze dye uptake, the geometric mean in FL4 (661/16 nm) was determined after incubation with DY-615 (λ_em_ = 641 nm), DY-630 (λ_em_ = 657 nm), and DY-635 (λ_em_ = 671 nm) and in FL3 (670 nm LP) after incubation with DY-736 (λ_em_ = 759 nm). Relative fluorescence units (RFUs) were determined from the difference between the averaged emissions of the investigated cells and co-incubated MACS beads. In contrast to the cell lines, the MNCs of CML patients were additionally labeled with CD33-PE and CD34-FITC. On the basis of the superimposed emission spectra of PE (λ_em_ = 575 nm) and FITC (λ_em_ = 520), the corresponding FL2 (585/342 nm) and FL1 (530/30 nm) were compensated with isotype controls (Table S5). Dye uptake in MNCs was evaluated in a FL1/FL2 plot. The geometric mean was determined in FL4, since only DY-630 and DY-635 were tested on MNCs of CML patients. Uptake behavior of DY-635 functionalized PLGA nanoparticles containing Nile Red as payload *DY-635[NP](NileRed)* was analyzed and compared to non-functionalized nanoparticles *[NP](NileRed)* in K562 cells and MNCs of CML patients. In contrast to MNCs CD34-separated bone marrow cells of five CML patients were labeled with CD38-APC and CD26-FITC after incubation with *[NP](NileRed)* or *DY-635[NP](NileRed).* Given the different fluorescence spectra of DY-635 and Nile Red (λ_em_ = 579 nm), the analyses were performed with the FL2/FL4 plot. Due to Nile Red as payload of both nanoparticle constructs, FL2 was used to quantify the nanoparticle uptake. The different Nile Red concentrations within *[NP](NileRed)* and *DY-635[NP](NileRed)* were taken into account by calculating a conversion factor using a derived linear regression equation. Therefore, a factor 1.10 higher fluorescence intensity in the channel FL2 was found for the 1.49-fold higher Nile Red concentration in *DY-635[NP](NileRed)* compared to *[NP](NileRed)* (Table S4).

### Confocal Laser Scanning Microscopy

Adherent HepaRG cells that were already differentiated on coverslips with a density of 2 × 10^5^ cells per well in 12-well plates were incubated with 1 mL of dye for 5 min at 37°C or 4°C and fixed by adding 2 mL of 10% formalin solution for 20 min at RT. After three washes with D-PBS, the cells were incubated with 500 μL of 0.1% Triton X-100/D-PBS solution for 10 min at RT and washed again. To each well, 1.5 μL phalloidin and 1 μL DAPI II (4′,6-diamidino-2-phenylindole) counterstain in 100 μL of D-PBS were added and incubated for 1 h at RT. After dye application on suspension cell lines, cytoskeleton staining was performed using the same procedure. Cells were transferred to slides by centrifugation via cytospine (Rotofix 32, Hettich) with 35 × *g* for 5 min at RT to obtain 2.5 × 10^5^ cells per slide. Slides dried overnight in the dark at RT. Microscopic evaluation was performed with the Laser Scanning Microscope LSM 510 META (Carl Zeiss, Jena, Germany) and ZEN 2009 software (Carl Zeiss).

### RNA Extraction and cDNA Synthesis

After hypotonic red cell lysis from at least 20 mL peripheral blood or bone marrow total RNA was extracted using a commercially available kit (innuPREP RNA Mini Kit, Analytik Jena AG, Jena, Germany) according to the manufacturer’s instructions. cDNA was synthesized using oligo(dt)-primer, dNTP mix (Fermentas), random hexamer primers (Thermo Fisher Scientific), reaction buffer (5 × First Strand Buffer, Invitrogen), DTT (Invitrogen), RNaseOut Recombinant (40 U mL^-1^ Ribonuclease Inhibitor) and M-MLV (Moloney Murine Leukemia Virus) reverse transcriptase (Invitrogen) as described elsewhere.^38^

### Quantitative Real-Time PCR

PCR primers for genes encoding carrier proteins were designed using Ensembl Genome Browser for sequence location (Table S6). Quantitative real-time PCR was performed using commercially available master mix (LightCycler 480 SYBR Green I Master Mix, Roche Diagnostics, Penzberg, Germany) according to the manufacturer’s instructions. Samples were run on 96-well plates in triplicates and gene expression was normalized using ΔC_t_ method against β-glucuronidase (β-GUS). Gene expression level of suspension cell lines was compared to that of HepaRG cells using the 2^-ΔΔCt^ method.

### RNA Interference

Knockdown of carrier protein gene expression was performed by nucleofection of siRNA in K562 cells. Therefore, a commercially available kit (Cell Line Nucleofector Kit V, LONZA/Amaxa, Basel, Switzerland) was used according to the manufacturer’s instructions regarding K562 cells. 30 pmol OATP1B3 siRNA and OCT1 siRNA (Table S7) were transfected in 1 × 10^6^ K562 cells each using program T-003 of the nucleofector (nucleofector II device, LONZA). Analogously, 30 pmol scrambled siRNA were transfected as a negative control. Knockdown experiments were performed in duplicate.

### Statistics

Comparison of two groups was performed with the two-tailed t test for independent samples. To compare the means of more than two groups, the one-way ANOVA was used. The degree of a linear relationship between two interval-scaled groups was determined using Pearson correlation. The significance level was 5%. Analysis and graphical presentation of data was done using SigmaPlot 13.0 software (Systat, San Jose, CA, USA). Data were presented using mean values with their standard deviation.

### Data Sharing Statement

For original data, please contact philipp.ernst@med.uni-jena.de.

## Author Contributions

Conceptualization, M.B., A.H., and P.E.; Methodology, P.E., J.H.C., A.T.P., M.F., V.G., and C.G.; Investigation, P.E., A.T.P., and J.W.; Writing, P.E., A.T.P., J.W., C.E., and J.H.C.; Funding Acquisition, P.E., A.H., and M.B.; Resources, A.T.P., T.E., U.S.S., M.L., C.E., M.B., C.G., and J.H.C.; Supervision, J.H.C., M.B., and A.H.

## Conflicts of Interest

M.L. and M.B. are shareholders of SmartDyeLivery GmbH. The other authors declare no competing interests.

## Supplemental Information

Document S1. Figures S1–S3 and Tables S1–S7

Document S2. Article plus Supplemental Information
