# Supplementary material for: Polymethine Dye-Functionalized Nanoparticles for Targeting CML Stem Cells
Source: Mol Ther Oncolytics. 2020 Jul 25;18:372–81. doi: 10.1016/j.omto.2020.07.007 (PMC7452122; doi:10.1016/j.omto.2020.07.007)
Supplement: Document S1. Figures S1–S3 and Tables S1–S7 [file mmc1.pdf]

OMTO, Volume *18*

Supplemental Information

Polymethine Dye-Functionalized Nanoparticles

for Targeting CML Stem Cells

Philipp Ernst, Adrian T. Press, Mike Fischer, Vivien Günther, Christine Gräfe, Joachim H. Clement, Thomas Ernst, Ulrich S. Schubert, Jana Wotschadlo, Marc Lehmann, Christoph Enzensperger, Michael Bauer, and Andreas Hochhaus

Supplemental Data

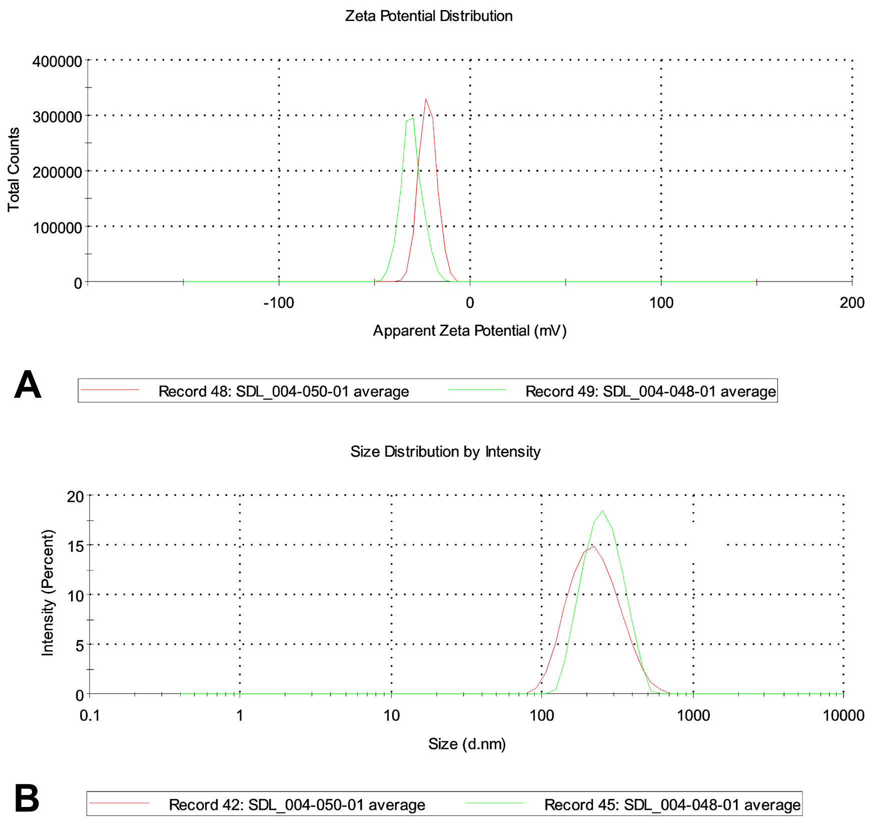

Figure S1. Zeta potential and size distribution of nanoparticle suspensions SDL-004-050-01 and SDL-004-048-01. (A) The graph shows surface charge of SDL_004-050-01 (red line; - 22 mV) and SDL_004-048-01 (green line; -31mV). The average results from three individual values from zeta potential measurements with a Zetasizer Nano ZS. (B) The graph shows the size distribution of SDL_004-050-01 (red line; 207 nm) and SDL_004-048-01 (green line; 241 nm) by intensity. Both nanoparticle suspension has a narrow size distribution and no aggregates. The average results from five individual measurements by dynamic light scattering with a Zetasizer Nano ZS. SDL_004-050-01 = *DY-635[NP](NileRed)*. SDL_004-048-01 = *[NP](NileRed)*.

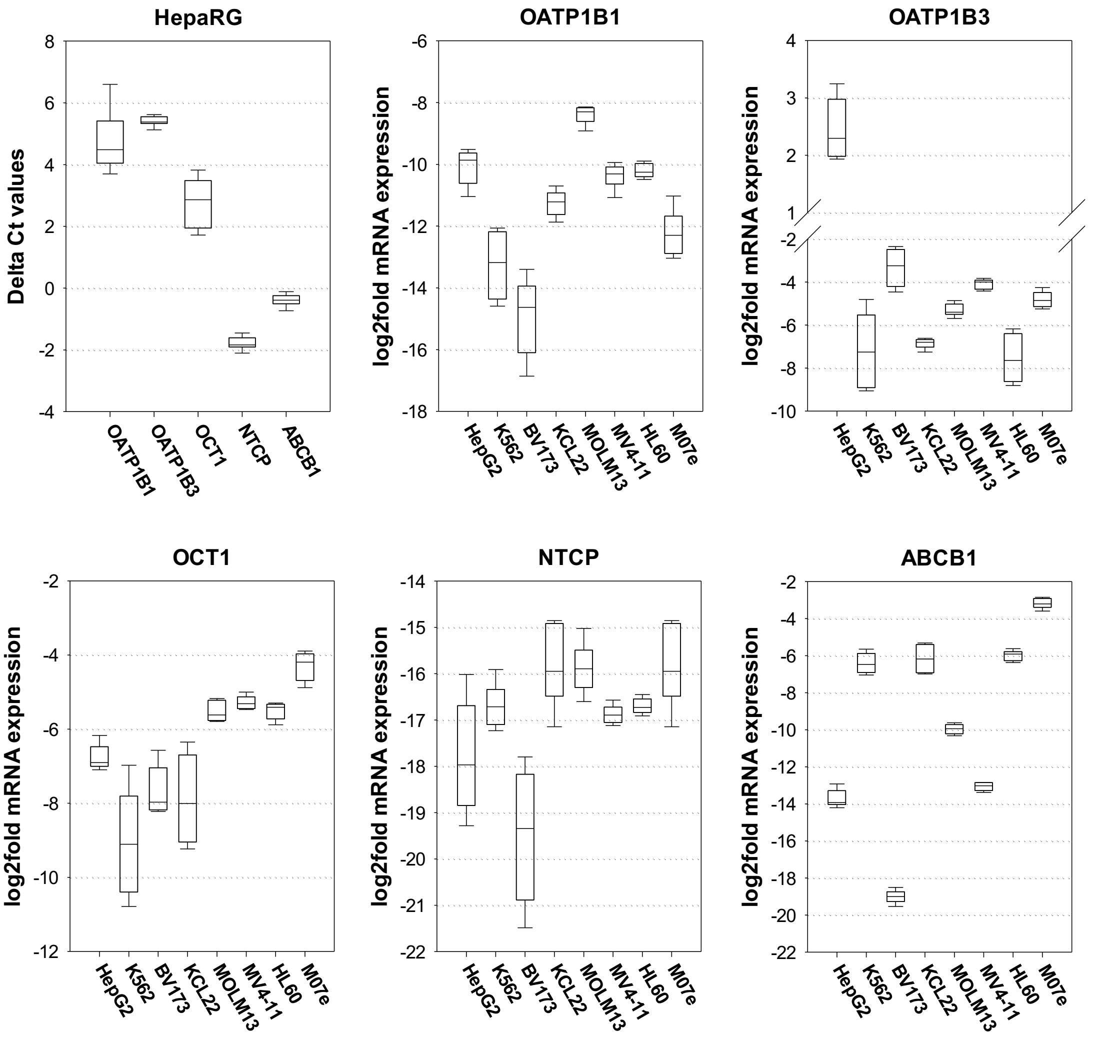

Figure S2. Quantitative Real-Time PCR measurements of mRNA expression of carrier proteins in different cell lines. Calculation of log2fold mRNA expression of carrier proteins was carried out with reference to the delta Ct values of the mRNA of the carrier proteins of the HepaRG cells. Delta Ct values refer to β-glucuronidase (β-GUS). Box plots with median and standard deviation are shown.

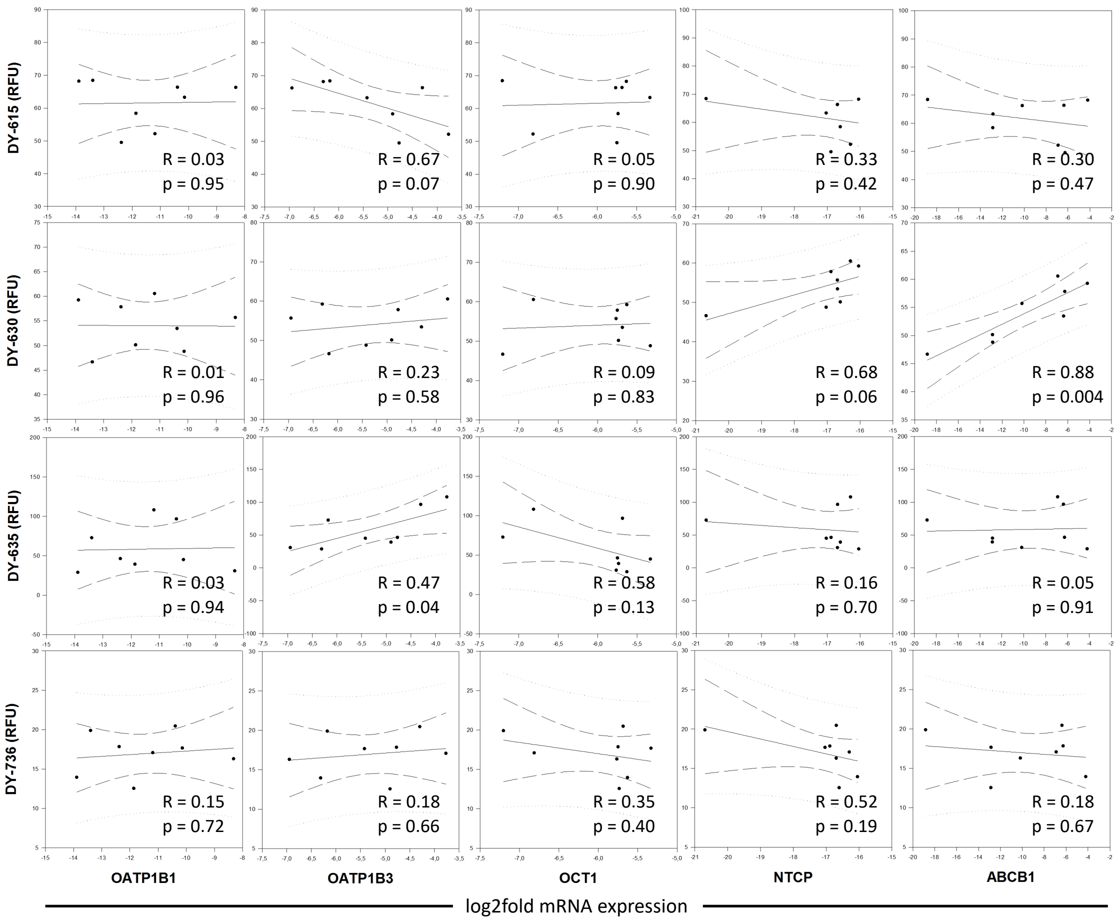

Figure S3. Correlation between dye uptake and mRNA expression of carrier proteins in leukemia cell lines. Pearson correlation of the RFU of leukemia cell lines after incubation with in each case 100 nM DY-615, DY-630, DY-635, and DY-736 with the log2-fold mRNA expression of carrier proteins OATP1B1, OATP1B3, OCT1, NTCP, and ABCB1 of the respective cell line. N = 8. R = Correlation coefficient. p = Probability of error.

Supplemental Materials

Table S1. Chronic myelogenous leukemia patients investigated

**Patient**	**Material**	**Age (years)**	**BCR-ABL1 transcript type**	**Sex**	**Patient**	**Material**	**Age (years)**	**BCR-ABL1 transcript type**	**Sex**
**#1**	PB	53	b2a2	m	**#16**	PB	49	b3a2	f
**#2**	PB	49	b3a2	f	**#17**	PB	32	b3a2	m
**#3**	PB	56	b2a2	m	**#18**	PB	51	b2a2/ b3a2	f
**#4**	PB	64	b3a2	m	**#19**	PB	74	b2a2	m
**#5**	PB	65	b2a2	f	**#20**	PB	31	b2a2	m
**#6**	PB	68	b3a2	m	**#21**	PB	75	b3a2	f
**#7**	PB	66	b3a2	m	**#22**	PB/ BM	55	b2a2	m
**#8**	PB	70	b3a2	f	**#23**	PB/ BM	72	b2a2	m
**#9**	PB	59	b2a2	m	**#24**	PB/ BM	58	b2a2	m
**#10**	PB	43	b3a2	m	**#25**	PB	30	b2a2/ b3a2	m
**#11**	PB	68	b3a2	f	**#26**	PB	59	b2a2	m
**#12**	PB	59	b3a2	m	**#27**	PB	50	b2a2	f
**#13**	PB	62	b3a2	m	**#28**	PB	52	b3a2	m
**#14**	PB	21	b3a2	f	**#29**	PB/ BM	19	b3a2	m
**#15**	PB/ BM	42	b3a2	f	**#30**	PB	52	b2a2/ b3a2	m

PB – Peripheral blood, BM – Bone marrow, m – male, f – female

Table S2. Structural formula, molecular weight and wavelength of Polymethine dyes and Nile Red

Dye	Structural formula	Molecular weight	Wavelength	Company
DY-635-amine	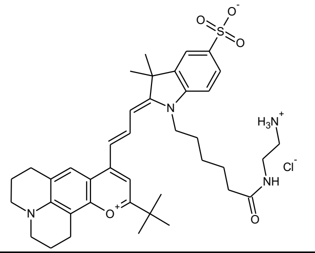	737.39 g mol^{-1} (as HCl salt)	λ_{abs} = 658 nm<br>λ_{em} = 695 nm	Dyomics GmbH, Jena, Germany
DY-630-amine	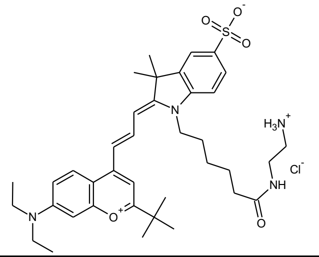	713.37 g mol^{-1} (as HCl salt)	λ_{abs} = 636 nm<br>λ_{em} = 657 nm	Dyomics GmbH, Jena, Germany
DY-615-amine	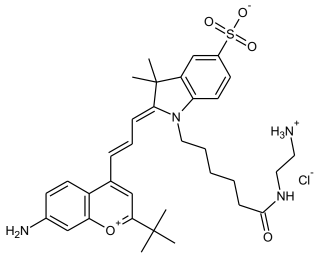	657.26 g mol^{-1} (as HCl salt)	λ_{abs} = 621 nm<br>λ_{em} = 641 nm	Dyomics GmbH, Jena, Germany
DY-736-amine	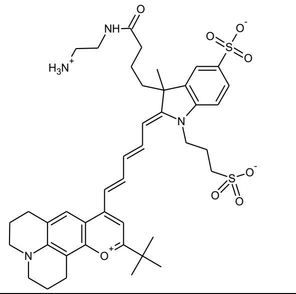	807.03 g mol^{-1} (zwitterionic)	λ_{abs} = 736 nm<br>λ_{em} = 759 nm	Dyomics GmbH, Jena, Germany
Nile Red	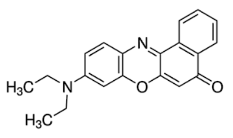	318.37 g mol^{-1} (as HCl salt)	λ_{abs} = 552 nm<br>λ_{em} = 638 nm	Sigma Aldrich, Darmstadt, Germany

Table S3. Hydrodynamic diameter (size), zeta potential and polydispersity index (PDI) of nanoparticle suspensions SDL-004-050-01 and SDL-004-048-01. *Poly(lactic-co-glycolic acid*) (PLGA), Nile red (NR)

Sample	Description	Size [nm]		PDI		Zeta potential [mV]	
			SD		SD		SD
SDL-004-050-01	PLGA-DY-365 + NR	207	± 1.718	0.119	± 0.038	-22	± 0.208
SDL-004-048-01	PLGA + NR	241	± 3.070	0.077	± 0.015	-31	± 0.586

Table S4. Concentration of Nile red (NR) and DY-635 of nanoparticle suspensions SDL-004-050-01 and SDL-004-048-01.

Sample	NR concentration [µg mL^{-1}] per 1 mg mL^{-1} nanoparticle concentration	DY-635 concentration [µg mL^{-1}] per 1 mg mL^{-1} nanoparticle concentration
SDL-004-050-01	0.730	0.649
SDL-004-048-01	0.489	0

Table S5. Antibodies and reagents for flow cytometry

Conjugates	Source	Specificity	Concentration	Company
Annexin V-APC			0.8 µL 100 $µL^{-1}$	ImmunoTools, Friesoythe, Germany
Propidiumiodid			0.4 µL 100 $µL^{-1}$	Sigma Aldrich, Taufkirchen, Germany
Anti-CD26-FITC	mouse	human	1:5	BD Biosciences, San Jose, USA
Anti-CD33-PE	mouse	human	1:5	BD Biosciences
Anti-CD34-FITC	mouse	human	1:5	BD Biosciences
Anti-CD38-APC	mouse	human	1:5	BD Biosciences
Mouse IgG2a control PE	mouse	unspecific	1:5	ImmunoTools
Mouse IgG1 control FITC	mouse	unspecific	1:5	ImmunoTools

Table S6. Sequences of PCR primers

Gene	Primer	Sequence in 5′→ 3′ direction	Annealing temperature
SLCO1B1 (OATP1B1)	fwd<br>rev	TGAACACCGTTGGAATTGC<br>TCTCTATGAGATGTCACTGGAT	60.2°C
SLCO1B3 (OATP1B3)	fwd<br>rev	GTCCAGTCATTGGCTTTGCA<br>CAACCCAACGAGAGTCCTTAGG	63.1°C
SLC22A1 (OCT1)	fwd<br>rev	GTGTGTAGACCCCCTGGCTA<br>GTGTAGCCAGCCATCCAGTT	63.1°C
SLC10A2 (NTCP)	fwd<br>rev	GGGACATGAACCTCAGCATT<br>CGTTTGGATTTGAGGACGAT	62.0°C
MDR1 (ABCB1)	fwd<br>rev	TCTGGAGGAAGACATGACCAGGTA<br>GGCACCAAAATGAAACCTGAATGT	61.0°C
β-GUS	fwd<br>rev	AGAAACGATTGCAGGGTTTCAC<br>CCGAGTGAAGATCCCCTTTTTA	62.0°C

Table S7. SiRNA for knockdown attempts

siRNA	Interrogated Sequence	Amount (per 100 µL cell suspension)	ID	Company
SLCO1B3 (OATP1B3) siRNA	NM_019844.3	30 pmol	s26262	Ambion, Thermo Fisher Scientific, USA
SLC22A1 (OCT1) siRNA	NM_003057.2	30 pmol	117134	Ambion, Thermo Fisher Scientific
All Stars negativ control scrambles siRNA		30 pmol	1027280	Qiagen N.V.
